# Metabolic syndrome is an independent risk factor for time to complete remission of fertility-sparing treatment in atypical endometrial hyperplasia and early endometrial carcinoma patients

**DOI:** 10.1186/s12958-022-01006-0

**Published:** 2022-09-05

**Authors:** Yingqiao Ding, Yuan Fan, Xingchen Li, Yiqin Wang, Jianliu Wang, Li Tian

**Affiliations:** 1 Reproductive Medical Center, Department of Obstetrics and Gynecology, Peking University People’s Hospital, Beijing, 100044 China; 2 Department of Obstetrics and Gynecology, Peking University People’s Hospital, Beijing, 100044 China

## Abstract

**Objective:**

Fertility-sparing treatment of atypical endometrial hyperplasia (AEH) and early endometrial carcinoma (EC) patients has recently emerged important social health topic. This study is designed to explore the risk factors for time to complete remission (CR) of fertility-sparing treatment in woman with AEH and early EC.

**Methods:**

A retrospective study was designed with clinical data from 106 patients admitted between January 2012 to December 2019. Univariate and multivariate logistic analysis were used to explore independent risk factors for time to CR. These factors were employed in receiver operator characteristic (ROC) curve and the decision curve analysis (DCA) to evaluate predictive accuracy of time to CR. Stratified analysis and interactive analysis was also performed for more in-depth perspective.

**Results:**

Univariate analysis showed that fasting blood glucose levels (FBG, OR = 1.6, 95%CI: 0.6–2.5, *P* = 0.020), metabolic syndrome (MetS, OR = 3.0, 95%CI: 1.1–5.0, *P* = 0.003), and polycystic ovary syndrome (PCOS, OR = 2.0, 95%CI: 0.5–3.4, *P* = 0.009) were associated with time to CR. Among these factors, multivariate analysis confirmed MetS (OR = 3.1, 95%CI: 1.0–5.2, *P* = 0.005) was an independent risk factor. The area under the ROC curve (AUC) of MetS was higher than FBG and PCOS (AUC = 0.723 vs 0.612 and 0.692). The AUC of FBG combined with PCOS was 0.779, and it was improved to 0.840 when MetS was included (*P* < 0.05). Additionally, MetS played different roles in time to CR in various groups. Moreover, we found high-density lipoprotein (HDL) and MetS had an interactive effect for time to CR.

**Conclusion:**

MetS is an independent risk factor for time to CR and should be taken seriously in fertility-sparing management of AEH and early EC patients.

**Supplementary Information:**

The online version contains supplementary material available at 10.1186/s12958-022-01006-0.

## Introduction

Endometrial carcinoma (EC) accounts for 7% of female malignancies in United States [1]. Most cases occur in postmenopausal women, but young women under the age of 40 account for 3.2 percent of EC patients and 57% of these cases being nulliparous [2]. As a precursor lesion, atypical endometrial hyperplasia (AEH) commonly evolves into EC. The standard treatment for EC is hysterectomy and bilateral salpingo-oophorectomy with or without lymphadenectomy, followed by adjuvant therapy according to risk factors. However, young patients usually have a strong desire to preserve fertility, especially for those without progeny. Such cases usually require fertility-sparing management using oral or uterine local progestin combined with GnRH-a or other regimen [3, 4], in conjunction with regular hysteroscopic biopsy.

The complete remission (CR) rate of fertility-sparing treatment of AEH and early EC patients aged 19–44 is 75% [5], which is influenced by various factors; and operative hysteroscopy, previous pregnancies, infertility, and megestrol acetate therapeutic regimen are associated with higher remission rates [5, 6]. Longer menstrual cycles and infrequent menstrual bleeding were identified as independent predictive factors for fertility-sparing treatment failure in AEH and early EC patients [7]. Since the risk factors of fertility-sparing treatment of AEH and early EC are not yet fully understood, it is of great significance to explore its risk factors.

Previous studies indicated that CR rate increased as conservative treatment duration was prolonged [8], and finally CR rate would reach the plateau after 12 months of treatment, at approximately 80% CR rate. Compared with CR rate, the recurrence rate increased continually over time for at least 5 years, which means the risk of recurrence elevated with the extension of fertility-sparing treatment duration [6]. Therefore, patients who haven’t reached CR after 12 months may not be suitable for continuous fertility-sparing treatment. For higher efficiency and safety of hormone therapy in AEH and early EC patients, we should not focus solely on CR, but also identify prognostic makers to predict time to CR.

However, risk factors for time to CR in fertility-sparing treatment of AEH and early EC patients have never been evaluated. This study aimed to clarify the risk factors for time to CR to guide the clinical selection of appropriate patients to receive fertility-sparing treatment.

## Materials and methods

### Patients

The clinical characteristics of patients who underwent fertility-sparing treatment for AEH and early EC from January 2012 to December 2019 were collected for retrospective analysis. The study was conducted in Peking University People’s Hospital (Beijing, China).

### Inclusion criteria

Patients who met the following criteria were included in our study: (I) Endometrial tissue obtained by dilation and curettage (D&C) confirmed that histological type was restricted to AEH and early EC; (II) Younger than 45 years old; (III) Patients had a strong desire to give birth and request for fertility-sparing treatment and finally reached CR with different time intervals; (IV) Patients diagnosed with early EC underwent pelvic magnetic resonance imaging (MRI) to exclude deep myometrial invasion or extra uterine lesions; (V) All included patients voluntarily agreed to sign informed consent for fertility-sparing treatment. Early EC was defined as endometrioid adenocarcinoma diagnosed with FIGO stage IA, grade 1.

### Variables and treatment protocol

The metabolic syndrome (MetS) criteria are put forward by the Chinese medical association diabetes branch which is defined as three or four of the following standards [9]: 1) BMI is greater than 25.0 kg/m^2^. 2) FBG is greater than 6.1 mmol/L and/or 2 h blood glucose (2 h BG) is greater than 7.8 mmol/L, and/or has been diagnosed with diabetes (DM). 3) Systolic/diastolic blood pressure was greater than 140/90 mmHg, and/or has been diagnosed with high blood pressure (HBP). 4) Blood Triglycerides (TG) is greater than 1.7 mmol/L, and/or blood high-density lipoprotein (HDL) < 1.0 mmol/L. We use the homeostasis model assessment-insulin resistance (HOMA-IR) to determine patient’s insulin resistant (IR) status. The HOMA-IR value is calculated as FBG (mmol/L) × fasting insulin (FINS, μU/mL)/22.5. Patients with DM or whose HOMA-IR ≥ 2.95 were considered as IR [10]. Other related factors were included, such as age, polycystic ovary syndrome (PCOS), gestation, parity, infertility, and histological type.

Patients were assigned by doctors to take 250/500 mg of medroxyprogesterone acetate (MPA) orally, or 160 mg of megestrol acetate (MA) orally, which is the first-line treatment for fertility-sparing treatment in AEH and early EC patients. An injection of GnRH-a every 28 to 30 days was applied in the case of no response to high-dose oral progesterone treatment.

### Outcomes

After the treatment, endometrial biopsy was performed by hysteroscopic biopsy to evaluate the treatment outcomes every 3 to 6 months. Ultrasound and endometrial biopsy were used to assess if the endometrium reached CR, which was defined as a normal endometrium without atypical hyperplasia. Time to CR is calculated from the date of starting fertility-sparing treatment to the date of achieving CR. Recurrence was defined as the reappearance of a lesion that had initially regressed following fertility-sparing treatment by hysteroscope. The information we needed was searched from electronical medical records. This study was approved by the Ethics Committees of Peking University People’s Hospital (No. 2020PHB063-01).

### Statistical analysis

The patients were classified by different time intervals to obtain CR. Data was presented as mean ± SD or proportions. Univariate and multivariate analyses were performed using the possible risk factors by a logistic regression to determine the likelihood ratio. The odds ratio (OR) was calculated along with 95% confidence intervals (95%CI). Stratified analysis was performed to further explore the independent risk factors effect on time to CR and the result was plotted as forest plot. Furthermore, ROC curve analysis and DCA were conducted to verify the validity of the prediction model. Interactive analysis was employed to determine any interactive effect between MetS and other metabolic factors. All of the analyses were performed with the statistical software packages of R version 3.4.3 (http://www.R-project.org, The R Foundation) and EmpowerStats (http://www.empowerstats.com, X&Y Solutions, Inc., Boston, MA). A two-sided significance level of *P* < 0.05 was considered statistically significant.

## Results

### Clinical characteristics of enrolled patients

**Fig. 1:**
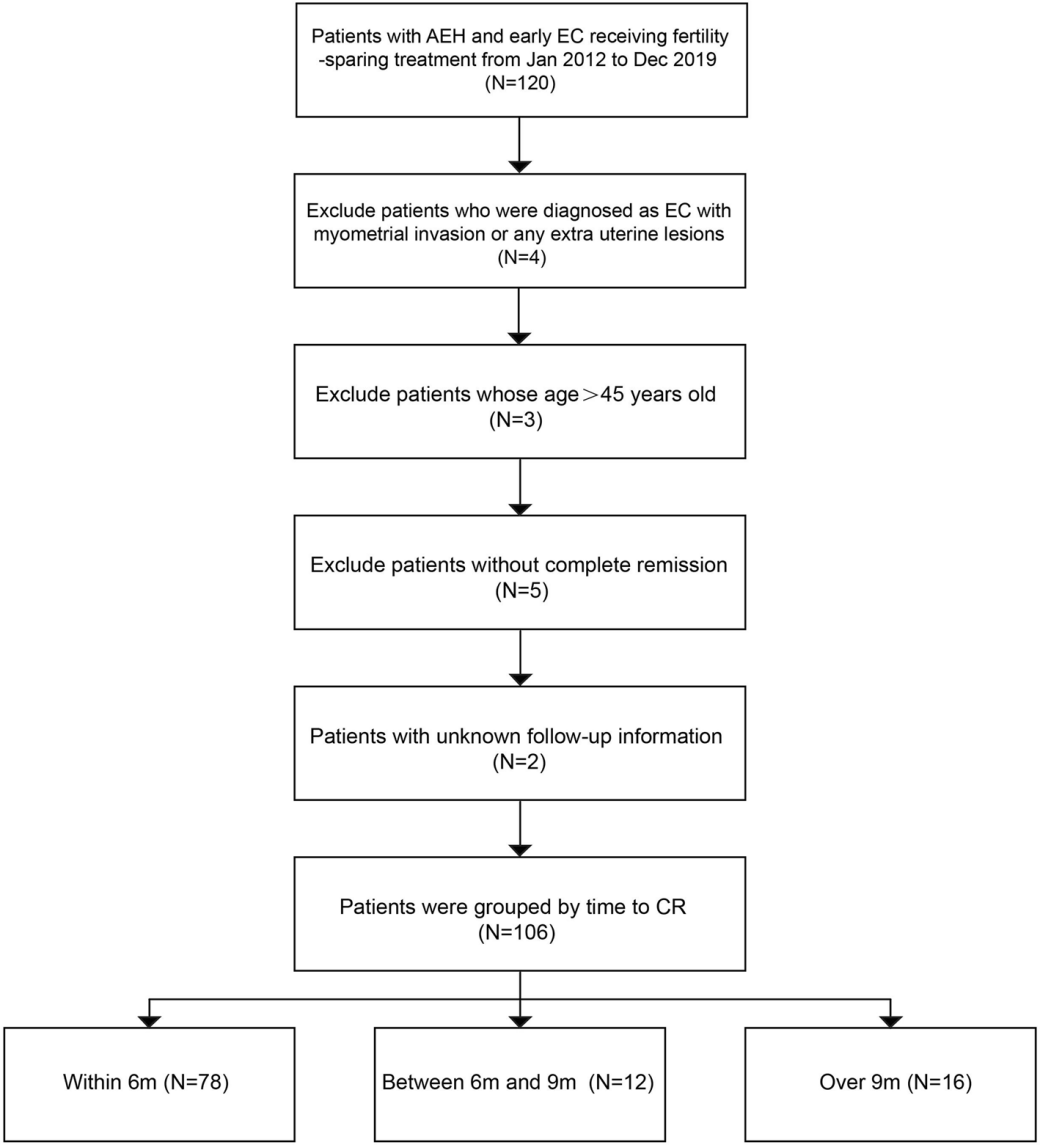
Flow chart of inclusion criteria and exclusion criteria. AEH: atypical endometrial hyperplasia; early EC: early endometrioid adenocarcinoma diagnosed with Grade1, Stage IA; CR: complete remission; 6 m: 6 months; 6-9 m: between 6 and 9 months; 9 m: 9 months

A total of 120 patients who underwent fertility-sparing treatment between January 2012 and December 2019 in Peking University People’s Hospital were reviewed during the study period. Of them, 59 patients with AEH and 47 early EC patients meet the study inclusion criteria (Fig. 1). Patients were grouped by time to CR: 78 patients reached CR within 6 months, 12 patients obtained CR between 6 and 9 months and 16 patients obtained CR over 9 months. Their clinical characteristics are summarized in Table 1. Baseline characteristics were comparable between groups, such as age, BMI, gestation and parity, infertility, complications, several metabolic index, histological type, oral medication regime, following time, and recurrence number. The HOMA-IR increased with longer time to CR with the value of 3.4 ± 2.7, 3.7 ± 2.6 and 4.8 ± 3.3, respectively (*P* = 0.212). There were 12.8%, 16.7% and 31.2% patients diagnosed with metabolic syndrome (MetS) in three defined groups respectively (*P* = 0.187), while polycystic ovary syndrome (PCOS) patients represented 41.0%, 33.3% and 81.2%, respectively (*P* = 0.008).

**Table 1:** Baseline characteristics of patients

	Time to CR			*P*-value
	< 6 m (*n* = 78)	6-9 m (*n* = 12)	> 9 m (*n* = 16)	
Age (years)	31.9 ± 4.3	31.2 ± 3.8	31.4 ± 4.1	0.833
BMI (kg/m^2^)	25.8 ± 4.4	27.1 ± 5.6	26.9 ± 3.9	0.561
HOMA-IR	3.4 ± 2.7	3.7 ± 2.6	4.8 ± 3.3	0.212
FBG (mmol/L)	5.0 ± 1.0	5.3 ± 1.5	7.0 ± 8.8	0.886
Serum insulin (μU/mL)	15.2 ± 10.5	16.1 ± 10.2	20.0 ± 14.7	0.455
HDL (mmol/L)	1.13 ± 0.02	1.13 ± 0.09	1.23 ± 0.16	0.442
Triglyceride(mmol/L)	1.61 ± 0.12	1.38 ± 0.19	1.90 ± 0.35	0.152
Following time (months)	35.2 ± 29.7	42.0 ± 23.2	34.9 ± 35.1	0.450
IR				0.547
No	41 (52.6%)	6 (50.0%)	6 (37.5%)	
Yes	37 (47.4%)	6 (50.0%)	10 (62.5%)	
DM				0.416
No	61 (78.2%)	8 (66.7%)	14 (87.5%)	
Yes	17 (21.8%)	4 (33.3%)	2 (12.5%)	
HBP				1.000
No	72 (92.3%)	11 (91.7%)	15 (93.8%)	
Yes	6 (7.7%)	1 (8.3%)	1 (6.2%)	
MetS				0.187
No	68 (87.2%)	10 (83.3%)	11 (68.8%)	
Yes	10 (12.8%)	2 (16.7%)	5 (31.2%)	
PCOS				**0.008**
No	46 (59.0%)	8 (66.7%)	3 (18.8%)	
Yes	32 (41.0%)	4 (33.3%)	13 (81.2%)	
Gestation				0.416
No	45 (57.7%)	9 (75.0%)	11 (68.8%)	
Yes	33 (42.3%)	3 (25.0%)	5 (31.2%)	
Parity				0.080
No	61 (78.2%)	12 (100.0%)	15 (93.8%)	
Yes	17 (21.8%)	0 (0.0%)	1 (6.2%)	
Infertility				0.908
No	42 (53.8%)	7 (58.3%)	8 (50.0%)	
Yes	36 (46.2%)	5 (41.7%)	8 (50.0%)	
Histological type				0.227
AEH	47 (60.3%)	6 (50.0%)	6 (37.5%)	
Early EC	31 (39.7%)	6 (50.0%)	10 (62.5%)	
Metformin				0.950
No	42 (53.8%)	7 (58.3%)	9 (56.2%)	
Yes	36 (46.2%)	5 (41.7%)	7 (43.8%)	
MPA250mg				0.448
No	31 (39.7%)	7 (58.3%)	6 (37.5%)	
Yes	47 (60.3%)	5 (41.7%)	10 (62.5%)	
MPA500mg				0.521
No	63 (80.8%)	8 (66.7%)	13 (81.2%)	
Yes	15 (19.2%)	4 (33.3%)	3 (18.8%)	
MA				0.148
No	68 (87.2%)	9 (75.0%)	11 (68.8%)	
Yes	10 (12.8%)	3 (25.0%)	5 (31.2%)	
GnRH-a				0.907
No	68 (87.2%)	11 (91.7%)	14 (87.5%)	
Yes	10 (12.8%)	1 (8.3%)	2 (12.5%)	
Recurrence				0.365
No	49 (62.8%)	8 (66.7%)	13 (81.2%)	
Yes	29 (37.2%)	4 (33.3%)	3 (18.8%)	

*CR* Complete remission, *6 m* 6 months, *6-9 m* Between 6 and 9 months, *9 m* 9 months, *BMI* Body mass index, *HOMA-IR* Homeostasis model assessment of insulin resistance-insulin resistance, *FBG* Fasting blood glucose, *HDL* High-density lipoprotein, *IR* Insulin resistance, *DM* Diabetes, *HBP* High blood pressure, *MetS* Metabolic syndrome, *PCOS* Polycystic ovarian syndrome, *AEH* Atypical endometrial hyperplasia, *early EC* Early endometrioid adenocarcinoma diagnosed with Grade1, Stage IA, *MPA* Oral medroxyprogesterone acetate, *MA* Oral megestrol acetate

### Risk factors associated with time to CR

Univariate analysis was conducted to investigate the risk and protective factors in relationship with time to CR (Table 2). Fasting blood glucose levels (FBG) (OR = 1.6, 95%CI: 0.6–2.5, *P* = 0.020), MetS (OR = 3.0, 95%CI: 1.1–5.0, *P* = 0.003), and PCOS (OR = 2.0, 95%CI: 0.5–3.4, *P* = 0.009) were identified as the risk factors associated with prolonged time to CR. No relationships were found between other clinical factors and time to CR (*P* > 0.05).

**Table 2:** Results of univariate analysis of time to CR in fertility-sparing treatment of AEH and early EC

	Time to CR	
	OR (95%CI)	*P*-value
Age (years)	-0.1 (-0.3, 0.1)	0.316
BMI (kg/m^2^)	0.2 (-0.0, 0.3)	0.054
HOMA-IR	0.1 (-0.1, 0.4)	0.377
FBG (mmol/L)	1.6 (0.6, 2.5)	**0.020**
Serum insulin (μU/mL)	0.0 (-0.0, 0.1)	0.608
HDL (mmol/L)	0.2 (-0.2, 0.5)	0.347
Triglyceride (mmol/L)	0.4 (-0.1, 0.6)	0.158
IR	0.3 (-1.2, 1.8)	0.657
DM	0.1 (-1.7, 1.9)	0.932
HBP	0.2 (-2.7, 3.0)	0.914
MetS	3.0 (1.1, 5.0)	**0.003**
PCOS	2.0 (0.5, 3.4)	**0.009**
Gestation	-0.7 (-2.3, 0.8)	0.355
Parity	-1.6 (-3.6, 0.4)	0.118
Infertility	0.1 (-1.4, 1.6)	0.885
Histological type	1.0 (-0.5, 2.5)	0.197
Metformin	-0.7 (-2.2, 0.8)	0.343
MPA250mg	0.7 (-0.8, 2.2)	0.363
MPA500mg	-0.1 (-1.9, 1.8)	0.923
MA	0.9 (-1.1, 2.9)	0.380
GnRH-a	-0.9 (-3.2, 1.3)	0.418

*CR* Complete remission, *BMI* Body mass index, *HOMA-IR* Homeostasis model assessment of insulin resistance-insulin resistance, *FBG* Fasting blood glucose, *HDL* High-density lipoprotein, *IR* Insulin resistance, *DM* Diabetes, *HBP* High blood pressure, *MetS* Metabolic syndrome, *PCOS* Polycystic ovarian syndrome, *MPA* Oral medroxyprogesterone acetate, *MA* Oral megestrol acetate

### Multivariate analysis for a prolonged time to CR

Multivariate analysis was performed to further explore the relationship between time to CR and FBG, MetS and PCOS. To determine if MetS is an independent risk factor for time to CR in the target population, we established two models adjusting for different confounding factors; Model I adjused for basic clinical characteristics including age, BMI, gestation, and parity; Model II added other risk factors including FBG and PCOS. Table S1 showed that MetS was an independent risk factor for prolonged time to CR in patients receiving fertility-sparing treatment, regardless of the used model (unadjusted model: OR = 3.0, 95% CI: 1.1–5.0, *P* = 0.003; Model I: OR = 2.5, 95%CI: 0.4–4.7, *P* = 0.022; Model II: OR = 3.1, 95%CI: 1.0–5.2. *P* = 0.005).

### Predictive accuracy of MetS for time to CR

**Fig. 2:**
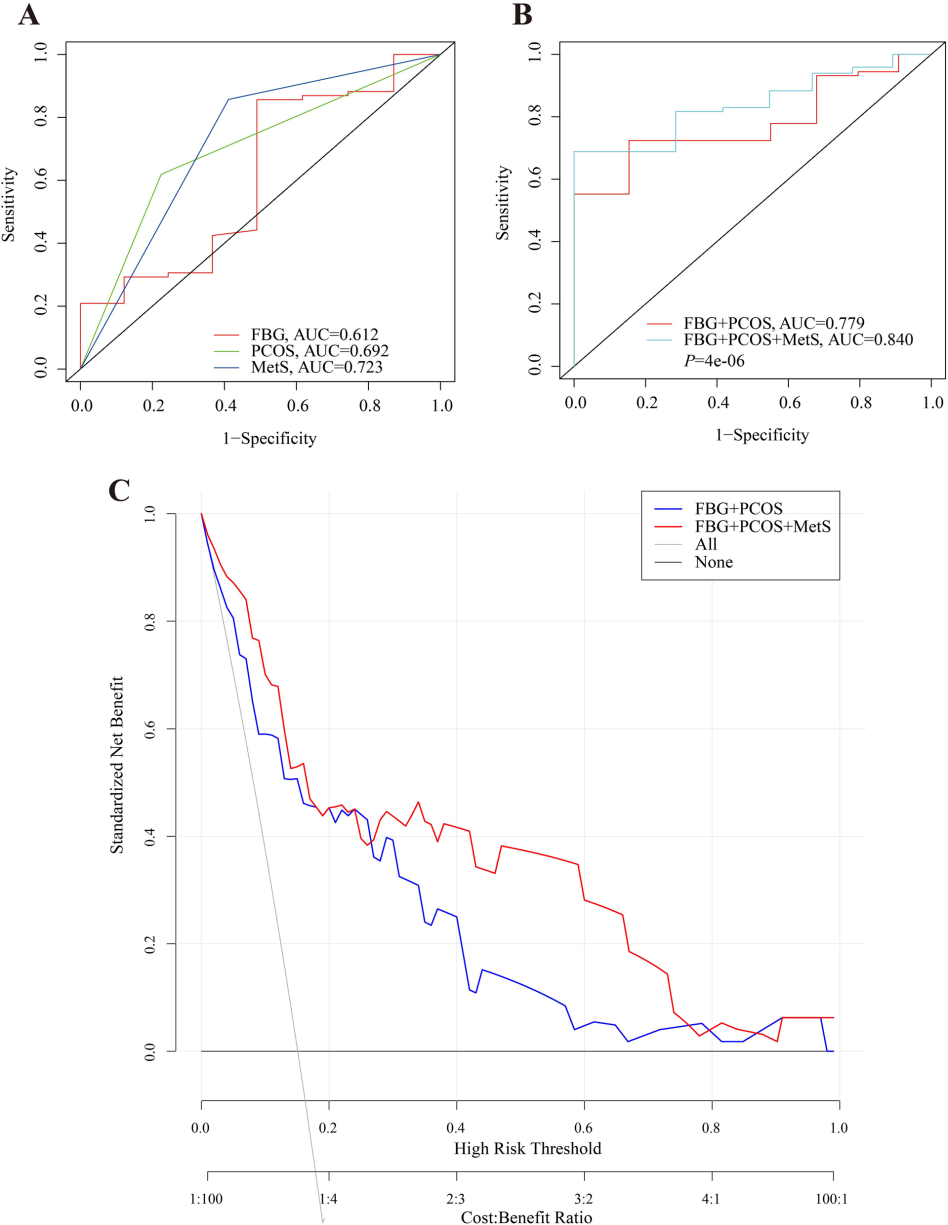
Predictive accuracy of different models. **A** Area under the receiver operating characteristic curves (AUCs) of FBG, PCOS and MetS respectively for the prediction of time to CR in fertility-sparing treatment patients; **B** Area under the receiver operating characteristic curves (AUCs) of FBG + PCOS and FBG + PCOS + MetS for the prediction of time to CR. **C** Decision curve analysis (DCA) of FBG + PCOS and FBG + PCOS + MetS for the prediction of time to CR in fertility-sparing treatment patients. FBG: fasting blood glucose; PCOS: polycystic ovarian syndrome; MetS: metabolic syndrome

To further estimate the predictive accuracy of different risk factors for time to CR after fertility-sparing treatment, we next performed ROC curve analysis. The AUC between 0.6 and 0.7, or above 0.7 was considered moderate or excellent performance respectively. When the predictive accuracy of the three risk factors was compared separately, the AUC of MetS was higher than FBG and PCOS (AUC = 0.723 vs 0.612 and 0.692, Fig. 2A). The AUC of FBG combining with PCOS was 0.779, and it reached to 0.840 when MetS was included (Fig. 2B). Therefore, MetS significantly improved the predictive value for time to CR. In order to further verify the predictive effectiveness of MetS, decision curve analysis (DCA) was employed (Fig. 2C). Predicted probability thresholds between 0% and nearly 80%, excluding a small range near 25%, showed a positive net benefit for fertility-sparing treatment patients when based on MetS compared to models based on FBG and PCOS alone. Therefore, these results suggested that MetS significantly increased the predictive accuracy of prolonged time to CR after fertility-sparing treatment in AEH and early EC patients.

### Stratified analysis of relationship between MetS and time to CR

**Fig. 3:**
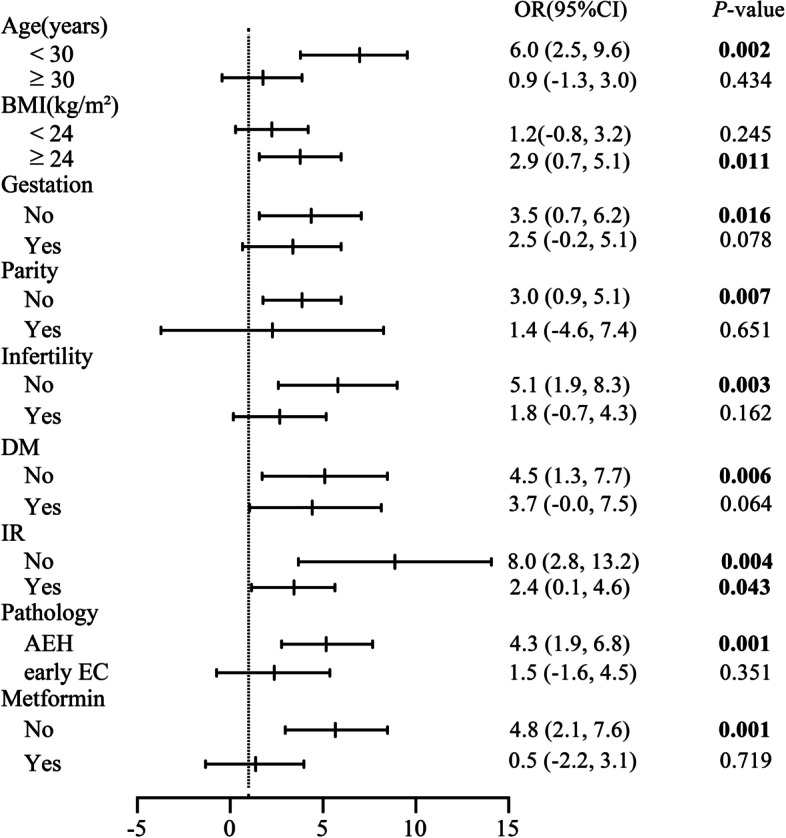
Forest plot for stratified analysis of time to CR in different groups of patients with MetS. BMI: body mass index; DM: diabetes; IR: insulin resistance; AEH: atypical endometrial hyperplasia; early EC: early endometrioid adenocarcinoma diagnosed with Grade1, Stage IA

To further explore the impact of MetS on time to CR in different groups of population, we next stratified the cohort for analysis (Fig. 3). MetS showed a favorable risk predictive value for time to CR in patients under the age of 30 (OR = 6.0, 95%CI: 2.5–9.6, *P* = 0.002) or overweight people with BMI ≥ 24 kg/m^2^ (OR = 2.9, 95%CI: 0.7–5.1, *P* = 0.011). Duration to CR of fertility-sparing therapy may be prolonged in patients that never been pregnant (OR = 3.5, 95%CI: 0.7–6.2, *P* = 0.016), nulliparity patients (OR = 3.0, 95%CI: 0.9–5.1, *P* = 0.007) and fertile patients (OR = 5.1, 95%CI: 1.9–8.3, *P* = 0.003) with MetS. In patients with the normal relevant metabolic index, without DM (OR = 4.5, 95%CI: 1.3–7.7, *P* = 0.006) or IR (OR = 8.0, 95%CI: 2.8–13.2, *P* = 0.004), the presence of MetS would extend the time to CR after fertility-sparing treatment. In addition, MetS had a negative impact on time to CR in AEH patients (OR = 4.3, 95%CI: 1.9–6.8, *P* = 0.001) comparing with early EC patients. MetS can also be used as a risk factor to predict time to CR in patients without using metformin regimen during fertility-sparing management (OR = 4.8, 95%CI: 2.1–7.6, *P* = 0.001). Therefore, MetS had a remarkable effect on time to CR during fertility-sparing treatment in various patients.

### The interactive effect between MetS and HDL

We further conducted the interactive analysis between MetS and HDL levels (Table 3). It indicated that middle HDL levels (1.0–1.2 mmol/L) had a risk predictive value for time to CR in patients diagnosed with MetS in Model I (OR = 18.9, 95%CI: 2.1–35.7, *P* = 0.0327). And in the middle HDL group, the risk of prolonged time to CR in MetS group significantly increased compared to the control group (OR = 31.3 vs OR = 24.3). In contrast, high HDL group did not show a higher risk of prolonged time to CR in patients with or without MetS (*P* > 0.05). Finally, the influence of MetS on time to CR decreased with elevated HDL levels in AEH and early EC patients (*P* for interaction < 0.05), suggesting that MetS performed diverse roles in patients with different HDL levels.

**Table 3:** Interactive analysis of MetS and HDL

MetS	HDL	Model I (OR, 95%CI) P	Model II (OR, 95%CI) P
No	Low	Ref	Ref
Yes	Low	0.2 (-4.7, 5.1) 0.9299	0.5 (-4.7, 5.8) 0.8464
No	Middle	12.6 (-2.7, 27.9) 0.1141	24.3 (0.9, 47.7) **0.0483**
Yes	Middle	18.9 (2.1, 35.7) **0.0327**	31.3 (6.2, 56.3) **0.0191**
No	High	-0.6 (-20.9, 19.7) 0.9547	0.3 (-21.4, 22.0) 0.9781
Yes	High	-0.4 (-21.9, 21.0) 0.9688	1.1 (-21.8, 24.1) 0.9224
P for interaction		**0.0261**	**0.0152**

*MetS* Metabolic syndrome, *HDL* High-density lipoprotein, *Model I Adjusted for* Age, BMI, Gestation, Parity, *model II Adjusted for* Age, BMI, Gestation, Parity, FBG, PCOS

## Discussion

Fertility-sparing treatment is usually applied for young patients with AEH and early EC who have a strong desire to retain future fertility potential. The administrations mainly include oral MPA or MA, GnRH-a, or Levonorgestrel Intrauterine device (LNG-IUD) [3, 4]. Although there is sufficient evidence of the efficacy and safety of fertility-sparing treatment, the selection of specific and appropriate patients is a problem that must be addressed.

Our previous studies suggested that the cumulative CR rates were 58.0%, 76.0% and 95.5% with the duration of treatment extending from 6 to 9 months to more than 9 months and sometimes longer, accompanied by the significantly increasing recurrence rate with 21.1%, 25.0% and 36.4% respectively [8]. This is consistent with the findings of Martin Koskas et al. Since CR rate increased over time but plateau, while recurrence rates don’t [6], blindly prolonging fertility-sparing treatment duration would increase the risk of recurrence. Our study focused on the factors that influenced the time to CR to identify patients suitable for extended conservative therapy.

We divided 106 patients with AEH or early EC into three groups based on the time intervals to achieve CR (< 6 m, 6-9 m, > 9 m). The basic characteristics of the three groups were roughly comparable, except for PCOS, which was more prevalent in groups with time to CR more than 9 months. Univariate analysis identified MetS, PCOS, and FBG as independent risk markers of prolonged time to CR and the effect of MetS was of particular concern. To further investigate the effect of MetS on time to CR, we performed a multivariate analysis. After adjusting for some basic confounding factors, such as age, BMI, gestation and parity, the risk effect of MetS on time to CR was still statistically significant. Additionally, the effect of MetS on time to CR was slightly elevated after adjusting for the other two risk factors PCOS and FBG. ROC curve analysis and DCA suggested that MetS significantly increased the predictive accuracy of longer time to CR after fertility-sparing management in AEH and early EC patients.

MetS is a cluster of components including IR, obesity, dyslipidemias, HBP and DM, with increasing incidence worldwide. Current viewpoints hold that MetS is closely associated with the occurrence and development of various cancers, which may be caused by the imbalance of estrogen and progesterone levels due to the status of metabolic imbalance. MetS is an independent prognostic factor of endometrial carcinoma and is closely related to tumor stage, grade, vascular invasion, tumor size, and lymph node metastasis [11]. The risk ratio of patients with three or more MetS symptoms increases around eightfold to diagnose EC, and is rising as the number of components increasing [12]. IR was usually indicated as the core symptom of MetS, which induced a chronic inflammatory state, manifested by excessive local expression of TNF-α, IL-6 and reactive oxygen species, resulting in persistent stimulation of endometrial cell hyperplasia [13]. Numerous studies have identified factors affecting CR rate in fertility-sparing therapy. In addition to the previously mentioned factors such as operative hysteroscopy, previous pregnancies, infertility, therapeutic regimens, longer menstrual cycles and infrequent menstrual bleeding [5–7], metabolism-related factors play a significant role in outcomes of these patients, such as PCOS, obesity and IR [14–16]. This is further supported by endometrial cancer association to hormone levels and metabolism [17]. Our results suggest that patients with MetS have longer time to CR. Therefore, AEH and early EC patients with MetS may be at risk of extended hormone treatment duration and require personalized fertility-sparing treatment.

To our knowledge, there are several diagnostic criteria for metabolic syndrome, such as the criteria proposed by World Health Organization (WHO), the European Group for the Study of Insulin Resistance (EGIR), the US National Cholesterol Education Program Adult Treatment Panel III (NCEP ATP III), and the International Diabetes Federation (IDF). The general principles in each definition are similar but the cutoffs and thresholds for the variables are somewhat different. There is no single unified diagnostic standard for MetS currently [18]. We referred to diagnostic criteria put forward by the Chinese medical association diabetes branch in 2004 [9]. However, different diagnostic standards for MetS can be applied for further study in the future.

A previous study indicated that the cumulative 16-week CR rate of early EC fertility-sparing treatment for PCOS patients was significantly lower compared to control group, while time to CR prolonged, and the relapse time after CR was shorter [14]. In our study, we found a significant increase in the proportion of PCOS patients who achieved CR with longer than 9 months therapy. Univariate analysis identified PCOS as a risk factor for time to CR, which indicated that a longer conservative therapy may be needed for PCOS patients to achieve CR. This phenomenon may be associated with progesterone resistance induced by low-grade chronic inflammation, insulin resistance and hyperandrogenemia in women with PCOS [14, 19].

After stratified analysis, we found that in women younger than 30 years old, MetS significantly prolonged the time to CR from fertility-sparing treatment, thus those patients may be at greater risk of relapse as the treatment continues. In overweight women with BMI ≥ 24.0 kg/m^2^, MetS was associated with 2.9-fold increased risk of prolonged time to CR. A previous study indicated that BMI ≥ 28.0 kg/m^2^ was an independent predictor of progesterone insensitivity [20], thus high BMI may be chosen as a risk marker for longer progestin treatment duration of AEH and early EC with MetS. In addition, MetS could predict longer time to CR in patients with AEH, but may not have a significant effect on time to CR in patients with early EC.

Interestingly, we noticed that patients with MetS who did not take metformin during conservative treatment were four times more likely to have a significantly longer time to reach CR. However, if they had ever taken metformin, the effect of MetS on prolonged time to CR would be reduced to 0.5 times. It was previously reported that metformin not only increased the early CR rate from 13.6% to 26.5% in patients with MetS during the fertility-sparing management of AEH and early EC, but also improved recurrence-free survival and overall survival for patients with early EC [21–23]. Mechanistically this may result from suppressed expression of Ki-67 via AMPK-dependent mTOR inhibition and extracellular signal-regulated kinase dephosphorylation by metformin [24, 25]. Alternatively, metformin may modulate inflammatory pathways and oxidative stress pathways by improving hyperglycemia and hyperinsulinemia, thereby reducing the stimulation of tumor cell growth [26]. Others may suggest that the MetS status probably has no impact on the efficacy of metformin together with MA for fertility-sparing treatment of AEH and early EC; but the implication was that regardless of the presence of MetS, treatment with metformin showed a higher CR rate than that with MA only, indicating the safety and efficacy of metformin [27]. Therefore, we suggest that treatment with metformin may shorten the time to CR in the course of fertility-sparing management for patients with MetS. This however required further validation with purpose-designed clinical trials.

We also observed an interaction between HDL levels and metabolic syndrome. In the middle HDL group, the risk of prolonged time to CR increased 24.3 times in the non-MetS group and 31.3 times in the MetS group. However, increase of HDL level, risk of MetS for time to CR was not significant. In other words, HDL could be regarded as a protective factor to alleviate the negative effect of MetS on time to CR. Many studies suggested that serum lipids were associated with EC, but the results were inconsistent. High HDL level had been shown to increase the risk of EC, primarily associated with non-endometrioid endometrial cancer [28], while other studies held the contrary view that HDL was unrelated to the elevated risk of EC [29]. Therefore, the specific role of HDL in the EC patients remains unclear, nor was it clear in fertility-sparing patients. In the general population, HDL levels represented as a J-type dose response manner associated with cancer mortality. It means that very high and very low HDL levels were related to elevated mortality [30]. This challenged the conventional view that the higher HDL level was better. As for our study, HDL levels in most of the patients were below 2.0 mmol/L, located on the descending part of the J-type curve, which may partially explain why high HDL levels improved outcomes of fertility-sparing treatment in the patients with MetS. Further research is needed to explore the protective mechanism of HDL on the outcome of fertility-sparing treatment of AEH and early EC patients.

To the best of our knowledge, this is the first study designed to screen risk factors for time to CR in AEH and early EC patients receiving fertility-sparing therapy, which is conducted with a large sample size and comprehensive analysis. However, there are some limitations in our study. This is a retrospective study and the factors which included in our analysis are mainly classic clinicopathologic factors. In recent years, novel molecular classification and molecular markers have become a hot research topic in endometrial cancer. But limited literature has explored their value in predicting the treatment response for fertility-sparing treatment in AEH and early EC patients. Loss of PTEN expression does not seem to predict the efficacy of fertility-sparing therapy [31]. Mismatch repair status could be used as a predictive biomarker for better treatment response of hormone treatment [32]. The identification of these biomarkers may improve clinical outcomes and promote the management of fertility-sparing treatment, with greater potential research value. Unfortunately, because of the retrospective nature of our data, the effect of molecular classification and other molecular markers on time to CR has not been clarified in our study. Future studies are needed to confirm the value of specific molecular predictors in fertility-sparing treatment. Therefore, a prospective and randomized controlled trial with large-sample size is required to validate the outcome of AEH and early EC patients with MetS undergoing fertility-sparing management, and to guide clinical decision-making.

## Conclusion

Our results suggest that MetS is a risk factor for prolonged time to CR in fertility-sparing management of AEH and early EC patients. The duration of treatment to obtain CR can be predicted independently by MetS or in combination with FBG and PCOS. The predictive value of MetS status is greater in young, overweight patients and AEH histological type. Additionally, metformin may have potential benefits to shorten time to CR in patients with MetS, but well-designed clinical trials are needed to shed further light.

## Supplementary Information

**Additional file 1:** **Table S1.** Logistic regression models evaluating the relationship between MetS and time to CR in fertility-sparing treatment of AEH and early EC.

## Data Availability

The datasets used and/or analyzed during the current study are available from the corresponding author on reasonable request.
