# Supplementary material for: Metabolic syndrome is an independent risk factor for time to complete remission of fertility-sparing treatment in atypical endometrial hyperplasia and early endometrial carcinoma patients
Source: Reprod Biol Endocrinol. 2022 Sep 5;20:134. doi: 10.1186/s12958-022-01006-0 (PMC9442985; doi:10.1186/s12958-022-01006-0)
Supplement: Supplementary file 1 — Additional file 1: Table S1. Logistic regression models evaluating the relationship between MetS and time to CR in fertility-sparing treatment of AEH and early EC. [file 12958_2022_1006_MOESM1_ESM.docx]

Table S1 Logistic regression models evaluating the relationship between MetS and time to CR in fertility-sparing treatment of AEH and early EC

| Exposure | unadjusted (OR, 95%CI) *P* | Model I (OR, 95%CI) *P* | Model II (OR, 95%CI) *P* |
| --- | --- | --- | --- |
| MetS |  |  |  |
| No | 1.0 | 1.0 | 1.0 |
| Yes | 3.0 (1.1, 5.0) **0.003** | 2.5 (0.4, 4.7) **0.022** | 3.1 (1.0, 5.2) **0.005** |

Non-adjusted model adjusted model for none; Model I Adjusted for: Age, BMI, Gestation, Parity; model II Adjusted for: Age, BMI, Gestation, Parity, FBG, PCOS.

MetS: metabolic syndrome; HR: hazard ratio; CI: confidence interval
